# Anatomical distribution of hemorrhoidal piles in advanced disease: clinical insights and correlations

**DOI:** 10.1007/s10151-025-03184-6

**Published:** 2025-07-22

**Authors:** İ. Osmanov, E. Ergüder, J. Ahmadov, C. Ersak, S. Leventoğlu, B. B. Menteş

**Affiliations:** 1 Department of Surgery, Ufuk University Dr. Rıdvan Ege Hospital, Ankara, Turkey; 2 Department of Surgery, Ankara Etlik City Hospital, Ankara, Turkey; 3 Proctology Unit, Ankara Memorial Hospital, Ankara, Turkey; 4 Department of Surgery, Gazi University Faculty of Medicine, Ankara, Turkey; 5 Department of Surgery, Kızılcahamam State Hospital, Ankara, Turkey

**Keywords:** Hemorrhoids, Anorectal disease, Surgical treatment, Proctology

## Abstract

**Background:**

A precise evaluation of the positional distribution of hemorrhoidal piles has not been distinctly conducted. We hypothesized that the distribution of hemorrhoidal piles follows a predictable anatomical pattern influenced by disease duration and recurrence.

**Methods:**

Our retrospective study analyzed the demographic data, surgical records, operative photographs, previous treatments, and associated colorectal symptoms of patients who underwent invasive procedures for advanced hemorrhoidal disease (2020–2024).

**Results:**

Of the 171 patients (123 male; 71.9%; median age 41 ± 12.04 years, range 18–88), 35 had prior interventions (recurrent cases). The largest pile was most commonly in the left lateral quadrant (40.14%), followed by right posterior (31.97%), right anterior (23.47%), and atypical locations (4.42%). Left lateral predominance was significantly higher in primary cases than in recurrent cases (*p = 0.031).* Most patients had more than one pile (87.7%). Symptom duration positively correlated with pile number (Spearman's rho = 0.229, *p* = 0.013), but not with hemoroid grade (*p = 0.977)*. No significant differences in pile distribution were observed in patients with defecation disorders, labor history, or concomitant anal fissure (*p* > 0.05). Of the 48 patients with anal fissure had significantly shorter symptom duration compared to those without fissure (*p* = 0.011).

**Conclusion:**

The classical three-quadrant distribution is confirmed, with the left lateral pile being predominant in primary cases. The association between prolonged symptom duration and increased pile number offers novel insights, highlighting left lateral predominance in primary cases and its reduction in recurrence, enhancing clinical understanding and management.

## Introduction

The understanding of hemorrhoidal disease has evolved over time. While the traditional varicose vein theory has been largely replaced, the sliding anal canal lining theory is now widely accepted [1]. This hypothesis suggests that hemorrhoids develop when the supportive tissues of the anal cushions undergo degeneration or deterioration [2, 3]. The concept of inferior displacement proposes that hemorrhoidal disease results from congestion, hypertrophy, and prolapse of the anal cushions. Additionally, increased resting and straining anal pressures have been linked to non-prolapsing hemorrhoids due to hypertensive anal cushions [4]. Chronic constipation and excessive straining exacerbate hemorrhoidal disease by raising intra-abdominal pressure, leading to hemorrhoidal plexus engorgement and the exertion of shearing forces on the anal cushions [5, 6].

Despite extensive pathophysiological studies, the reasons for hemorrhoids’ common anatomical locations remain unclear. Thomson [7] described three primary anal cushions at the left lateral, right anterior, and right posterior positions, with the right anterior cushion potentially more prone to prolapse due to defecatory forces. However, Ray-Offor and Amadi [8] reported multi-quadrant involvement in 72.5% of external hemorrhoid cases, with right posterior predominance. Given the clinical complexity and surgical relevance of advanced cases (grades 2–4), we aimed to investigate the anatomical distribution of hemorrhoidal piles and their associations with clinical characteristics. We hypothesized that pile distribution might follow a predictable anatomical pattern influenced by disease duration and recurrence.

## Patients and methods

### Patients

This study retrospectively analyzed individuals who underwent invasive hemorrhoidal procedures between July 2020 and February 2024. Medical records were reviewed for defecation disorders, coexisting conditions such as anal fissure, recurrence, prior proctologic surgeries, and obstetric history. Except for patients treated with band ligation, all cases had pre- and postoperative photographs recorded by the surgical team. Surgical records and imaging were assessed to determine the precise locations of hemorrhoidal clusters.

**Fig. 1:**
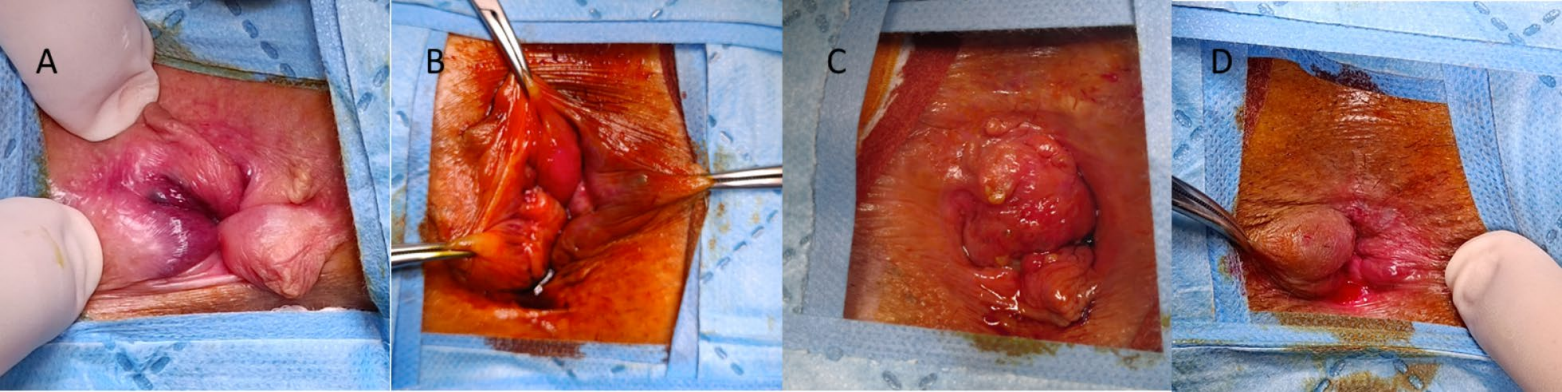
Examples of preoperative photographs. Four dominant piles (**A**), three dominant piles (**B**); two dominant piles (**C**); one dominant pile (**D**)

Only symptomatic grade 2–4 internal hemorrhoids (Goligher’s classification: grade 1, no prolapse; grade 4, irreducible prolapse) [9] with or without external components were included (Fig. 1). Patients diagnosed with newly developed symptomatic grade 1 hemorrhoids were managed conservatively through dietary and lifestyle modifications, as well as topical or oral medications, and were therefore excluded from the study. The number and anatomical distribution of hemorrhoids were evaluated according to the type of procedure performed and associated symptoms.

Recurrent cases were defined as patients who had previously undergone surgical or any invasive treatment, including rubber band ligation. Patients treated solely with conservative measures (e.g., dietary advice, topical agents) were not excluded. As a result of the retrospective nature of the study and the absence of detailed operative documentation from prior interventions, it was not possible to ascertain the exact anatomical location of previously treated piles. Therefore, the term “recurrent” in this study refers to the clinical need for re-intervention after a prior procedural treatment, rather than confirmed anatomical recurrence in a specific quadrant. The study was approved by Memorial Hospital Ethics Committee (Approval No. 2024-4, 27.09.2024).

### Procedures

**Fig. 2:**
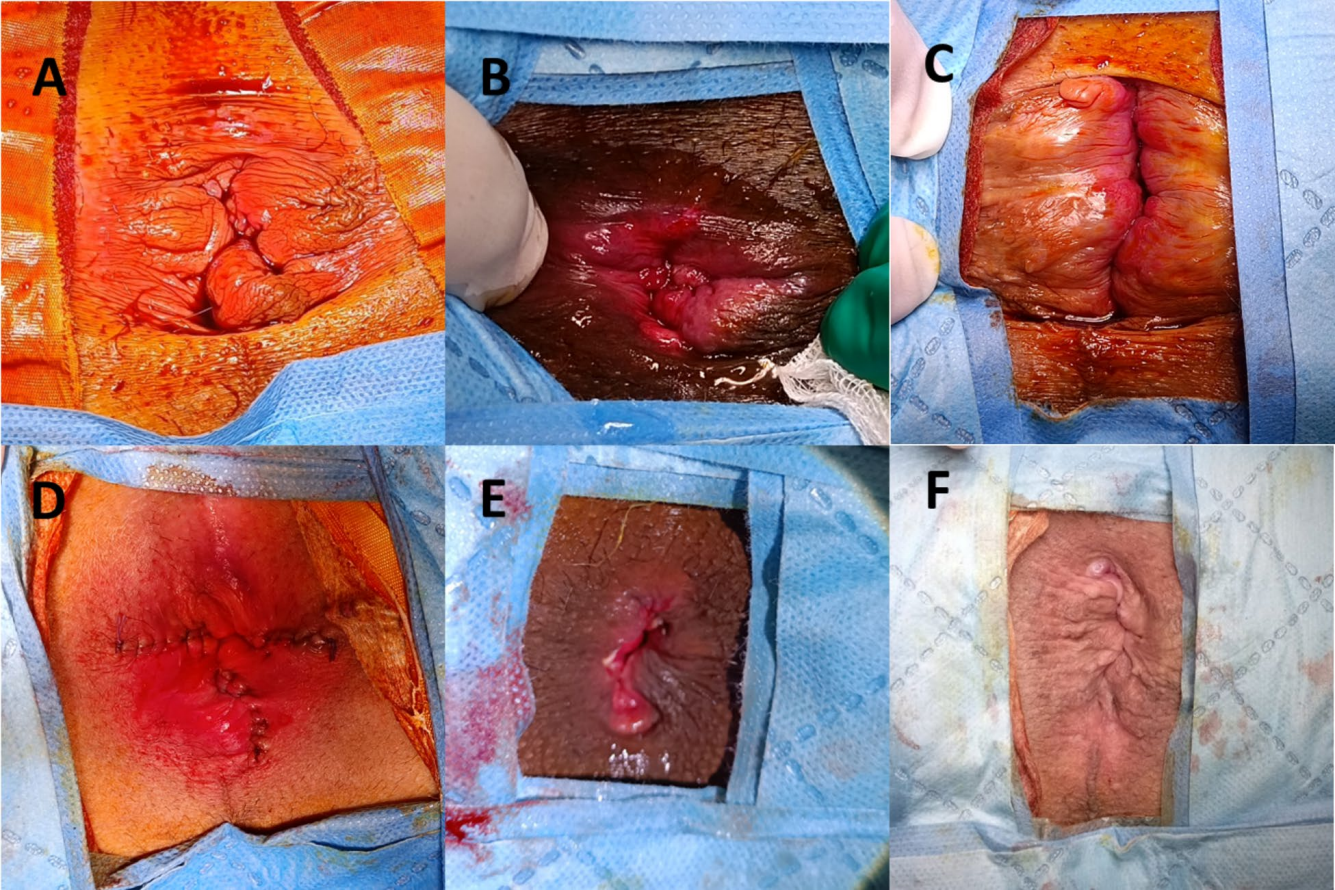
Some pre–postoperative photographs of the surgical procedures performed. Ferguson hemorrhoidectomy for three dominant piles (**A** preoperative, **D** postoperative). Laser ablation of hemorrhoids (**B** preoperative, **E** postoperative). Operative view of THD (**C** preoperative, **F** postoperative)

We performed Ferguson hemorrhoidectomy, rubber band ligation (RBL), transanal hemorrhoid dearterialization (THD), and/or laser ablation of hemorrhoids (LAH) depending on the grade of hemorrhoids and the patients’ complaints. Some patients underwent combinations of these techniques as a result of the heterogeneity of their piles (Table 1, Fig. 2). All patients were operated on by or under the supervision of a single highly experienced proctologist (BM).

**Table 1:** Distribution of patients according to surgery performed

Operation	*N* (%)	Grade			
		1	2	3	4
Ferguson hemorrhoidectomy	65 (38)	–	–	53	12
Rubber band ligation	33 (19.3)	–	32	1	–
Laser coagulation	4 (2.3)	–	–	4	–
THD	3 (1.8)	–	–	–	3
Ferguson—RBL	63 (36.8)	–	–	60	3
Ferguson—Laser	2 (1.2)	–	–	2	–
Ferguson—THD	1 (0.6)	–	–	1	–

* THD* transanal hemorrhoid dearterialization, *RBL* rubber band ligation, *LAH* laser ablation of hemorrhoids

### Statistical analysis

Continuous variables were summarized as medians with ranges. Categorical variables were expressed as frequencies and percentages.

The normality of continuous variables was assessed using the Shapiro–Wilk test. Between-group comparisons of categorical variables were performed using the chi-square test or Fisher’s exact test, as appropriate. Continuous variables were compared using the Mann–Whitney *U* test, based on distribution characteristics. Correlation analyses were performed using Spearman’s rank correlation coefficient to examine associations between symptom duration and factors such as the number of dominant hemorrhoidal piles. Additionally, bivariate comparisons of symptom duration were conducted across subgroups with and without defecation disorder and anal fissure, using the Mann–Whitney *U* test. All statistical analyses were conducted using SPSS version 23.0 (IBM Corp., Armonk, NY, USA), and a two-sided *p* value < 0.05 was considered statistically significant.

## Results

Of 171 patients (123 male; 71.9%; median age 41 ± 12.04 years, range 18–88), 35 (20.5%) had recurrent disease.

The distribution of hemorrhoid grades and quadrant involvement is presented in Table 2. The largest pile was most frequent in the left lateral quadrant (40.14%), followed by right posterior (31.97%), right anterior (23.47%), and atypical locations (4.42%). Left lateral predominance was higher in primary cases than in recurrent cases (*p* = 0.031). Stratifying patient groups with defecation disorders, concomitant anal fissure, or labor history and comparing these patient groups with the rest, no significant changes in the distribution of piles were noted (*p* > 0.05 for all comparisons), (Table 3).

**Table 2:** Distribution of hemorrhoid piles according to grade and involvement

Parameter	Category	Patients *n* (%)
Hemorrhoid grade distribution	Grade 2	32 (19)
	*Grade 3*	131 (71)
	Grade 4	8 (10)
Quadrant involvement	Single quadrant	21 (12)
	*Two quadrants*	83 (49)
	Three quadrants	64 (37)
	Four or more quadrants (circumferential involvement)	3 (2)
Dominant hemorrhoidal pile location*	*Left lateral*	117 (40)
	Right posterior	92 (32)
	Right anterior	70 (24)
	Atypical locations	13 (4)

The most frequent category for each parameter is in italics. Percentages were rounded to the nearest integer

*As some patients presented with multiple hemorrhoidal piles, the reported counts and percentages represent the total number of piles rather than the number of patients

**Table 3:** Anatomical distribution of hemorrhoidal piles stratified by etiological factors

Etiological factors	Status	Hemorrhoidal positions							
		Left lateral* n* (%)		Right anterior *n* (%)		Right posterior *n* (%)		Atypical *n* (%)	
		Present	Absent	Present	Absent	Present	Absent	Present	Absent
Defecation disorder	Absent	59 (38)	13 (8)	36 (23)	36 (23)	45 (29)	27 (17)	6 (4)	66 (47)
	Present	59 (38)	24 (16)	34 (22)	49 (32)	49 (37)	34 (22)	6 (4)	77 (48)
Anal fissure	Absent	85 (55)	26 (17)	53 (34)	58 (37)	69 (45)	42 (27)	8 (5)	103 (67)
	Present	33 (21)	11 (7)	17 (11)	27 (17)	25 (16)	19 (12)	4 (7)	40 (26)
Recurrence	Absent	100 (65)*	25 (16)	53 (34)	72 (47)	79 (51)	46 (28)	9 (6)	116 (75)
	Present	18 (12)	12 (8)	17 (11)	13 (8)	15 (10)	15 (10)	3 (2)	27 (17)
Labor history	Absent	7 (25)	1 (4)	3 (11)	5 (18)	3 (11)	5 (18)	1 (4)	7 (25)
	Present	12 (43)	8 (29)	9 (32)	11 (39)	12 (43)	8 (29)	1 (4)	19 (68)

Values are presented as number (%). Significant comparisons are highlighted with *p < 0.05. Since multiple hemorrhoidal piles may occur in a single patient, the values presented are not mutually exclusive and were not statistically compared across positions. Each etiological factor was independently cross-tabulated with pile location. Percentages were rounded to the nearest integer

A significant positive correlation was observed between symptom duration and pile number (*n* = 116, Spearman’s rho = 0.229, *p* = 0.013), but not with hemorrhoid grade (*p* = 0.977). Of the 48 patients with concomitant anal fissures, 70.8% exhibited two dominant piles, whereas the remaining had three dominant piles. Patients with anal fissures had a significantly shorter symptom duration compared to those without anal fissures (*p* = 0.011), (Table 4).

**Table 4:** Comparison of symptom duration between patients with and without anal fissures

	Status	Median symptom duration, months (min–max)	*p* value
Concomitant anal fissure	Absent (*n* = 89)	6 (0.25–240)	0.011
	Present (*n* = 27)	2 (0.25–24)	
Defecation disorder	Absent (*n* = 50)	6 (0.25–120)	0.612
	Present (*n* = 66)	5.5 (0.25–240)	

## Discussion

Advanced hemorrhoidal treatment poses challenges due to recurrence, complications, and postoperative morbidity [10–12]. Despite considerable research, the reasons for the common anatomical locations of hemorrhoids remain elusive. Our findings confirm that advanced hemorrhoidal piles predominantly adhere to the classical topographical distribution of the left lateral, right posterior, and right anterior cushions, with atypical locations in less than 5% of cases. This aligns with prior anatomical studies [7]; however, our data offer nuanced insights into pile distribution, diverging from some existing reports. Unlike Ray-Offor et al. [8], who reported right posterior predominance, we identified the left lateral cushion as the most affected site (40.14%). This discrepancy may reflect population-specific factors, referral patterns to a specialized unit, or differences in assessment methods (e.g., clinical vs. intraoperative mapping).

A key finding is the diminished left lateral predominance in recurrent cases compared to primary cases (*p* = 0.031). This may result from targeted interventions during initial treatment, which likely addressed the most symptomatic or prominent pile—commonly the left lateral quadrant. Consequently, the lower rate of left lateral involvement in recurrent cases may reflect effective prior treatment of that region. However, it is important to note that detailed data regarding the exact anatomical sites treated in previous interventions were not available, and this interpretation remains speculative. Despite this, the persistent involvement of the left lateral quadrant in some recurrent cases may still suggest an enduring anatomical predisposition. This dynamic shift suggets that treatment history modifies positional patterns—an observation not previously emphasized in the literature.

Our study also revealed multi-quadrant involvement in 87.7% of patients, exceeding the 72.5% reported by Ray-Offor et al. [8]. Conversely, 12.3% exhibited single-quadrant disease, challenging the assumption of uniform vascular cushion involvement [7, 13]. This heterogeneity underscores the complexity of hemorrhoidal anatomy and suggests that single-quadrant cases may represent earlier disease stages or distinct pathophysiological subsets warranting further investigation.

Most patients (81%) had grade 3 or 4 hemorrhoids with significant external components, contrasting with epidemiological data showing predominantly low-grade, asymptomatic disease [14]. This discrepancy likely stems from referral bias to our specialized proctology unit, skewing the cohort toward advanced cases and limiting generalizability to primary care settings. Positional predilection remained consistent across groups, showing no correlation with etiological factors such as defecation disorders, anal fissures, or obstetric history. This suggests that while these factors drive disease progression, they do not dictate anatomical distribution.

Notably, patients with concomitant anal fissures had shorter symptom durations (*p* = 0.011) and fewer dominant piles, likely due to heightened pain prompting earlier consultation. This aligns with a significant correlation between symptom duration and pile number (Spearman’s rho = 0.229, *p* = 0.013), but not grade (*p* = 0.977), indicating that pile multiplicity, rather than prolapse severity, reflects chronicity. This finding has diagnostic implications, as pile number could complement traditional grading in assessing disease burden.

Clinically, our data highlight the heterogeneity of hemorrhoidal piles, with patients presenting one, two, three, or more dominant piles of varying grades. Over one-third required multimodal interventions (e.g., excisional hemorrhoidectomy plus band ligation), underscoring the limitations of single-modality treatments like laser ablation. From a surgical perspective, this suggests that treatment plans should be tailored to pile number and distribution, not just grade, challenging the reliance on standardized techniques, as suggested by Elbetti et al. [15]. Dekker et al. and Elbetti et al. also concluded that Goligher’s classification should be revised with an international consensus. Despite our limitations we think that pile multiplicity may thus serve as an additional severity metric alongside Goligher’s classification [16, 17].

Limitations include the retrospective design, introducing selection and information biases. Conducted at a dedicated colorectal center, our study likely overrepresented advanced cases, reducing applicability to milder disease. Additionally, as a result of the retrospective design, detailed records of the exact anatomical sites treated in prior interventions were unavailable, limiting definitive interpretation of pile location patterns in recurrent cases. Although our sample (*n* = 171) is robust, multicenter prospective studies are needed to validate findings and enhance generalizability. Variability in surgical techniques and surgeon experience may also have influenced outcomes, a factor not controlled for in this analysis but critical for interpreting recurrence rates. Another notable limitation is the absence of validated patient-reported outcome measures (PROMs), such as the Hemorrhoid Disease Symptom Score (HDSS) and the Short Health Scale (SHS). Although our focus was on anatomical distribution and intraoperative findings, the inclusion of PROMs would have strengthened the assessment of symptom burden and its correlation with pile number and location. Future prospective studies should incorporate standardized PROM tools to better capture the patient perspective.

Future research should prioritize prospective designs to confirm the correlation between pile number and chronicity, using standardized intraoperative mapping to refine anatomical insights. Optimizing multimodal strategies for multi-pile, multi-grade disease could improve outcomes, guided by tailored patient selection criteria.

## Conclusion

Our findings suggest that (i) advanced piles do adhere to the well-known topography of left lateral, right posterior, and right anterior hemorrhoidal cushions; (ii) the left lateral predominance in our primary patients loses its power in recurrent cases; (iii) a positive correlation exists between the duration of hemorrhoid symptoms and the number of piles; (iv) the duration of complaints in patients with co-existing anal fissures is significantly shorter compared to those without anal fissures, and the number of significant piles is also smaller in this patient group; and (v) in a given patient, there may exist one, two, three, or more significant piles, and each may be of a different grade, requiring a combination of procedures.

## Data Availability

The datasets analyzed during the study are available from the corresponding author upon reasonable request.
